# A hidden gem Catenin-α-1 is essential for Chikungunya virus infection

**DOI:** 10.1128/spectrum.02485-23

**Published:** 2023-11-14

**Authors:** Sanchari Chatterjee, Bharat Bhusan Subudhi, Soma Chattopadhyay

**Affiliations:** 1 Infectious Disease Biology, Institute of Life Sciences, Bhubaneswar, Odisha, India; 2 Regional Centre for Biotechnology, Faridabad, Haryana, India; 3 School of Pharmaceutical Sciences, Siksha O Anusandhan Deemed to be University, Bhubaneswar, Odisha, India; Indian Institute of Science, Bangalore, Karnataka, India

**Keywords:** Chikungunya virus, Catenin-α-1, CHIKV-nsP2, co-immunoprecipitation

## LETTER

**Fig 1:**
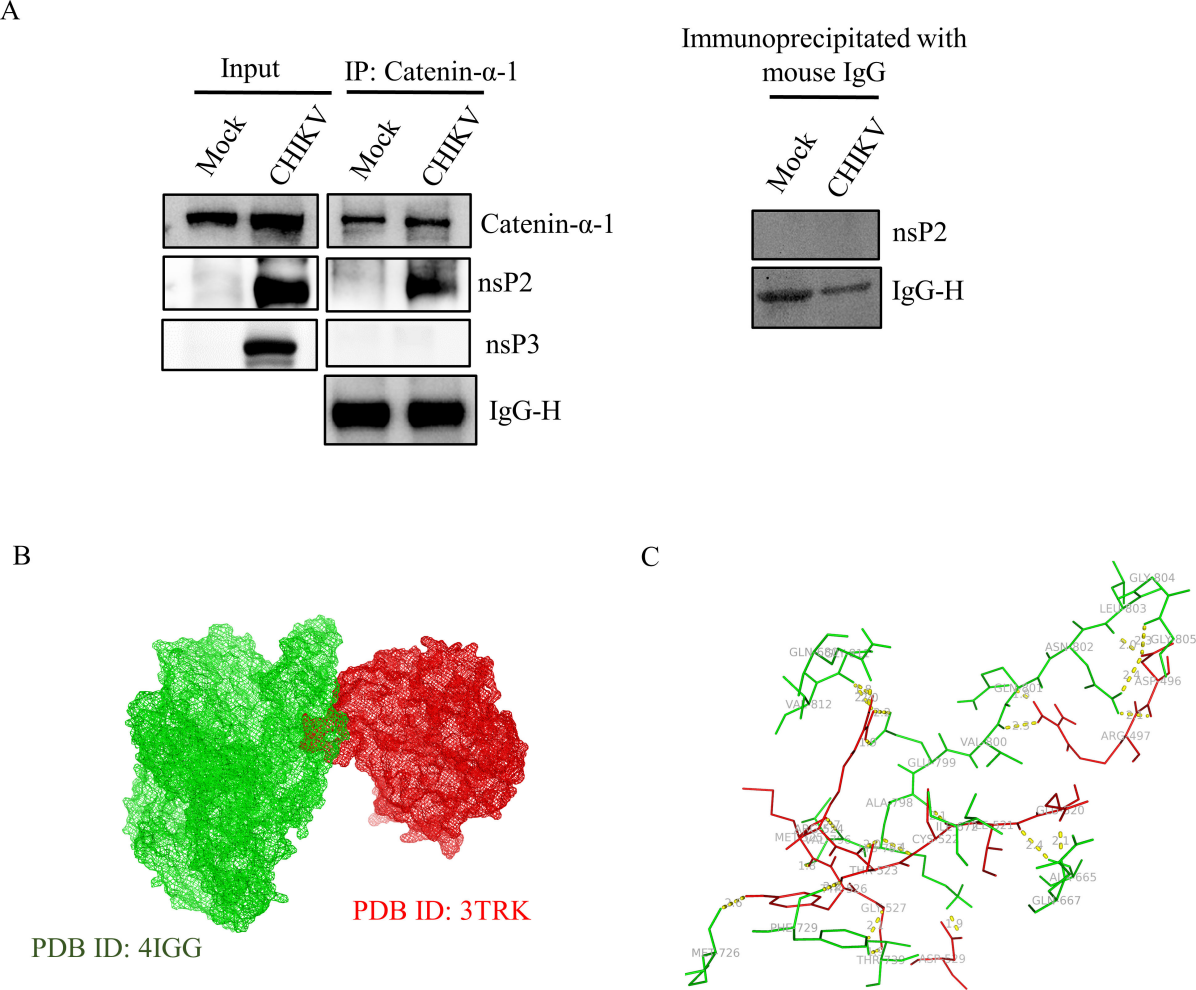
CHIKV-nsP2 interacts with the host Catenin-α-1 protein during CHIKV infection. Vero cells were infected with CHIKV and harvested at 6 hpi. The cell lysates were co-immunoprecipitated with the Catenin-α-1 antibody. (**A**) Western blot analysis depicting the levels of Catenin-α-1, nsP2, and nsP3 in the whole cell lysate (left), and co-immunoprecipitation analysis showing the interaction of the CHIKV-nsP2, nsP3, and Catenin-α-1 proteins (middle). Right panel represents the negative control, where normal mouse IgG was used to immunoprecipitate the protein complex and probed with the nsP2 antibody. (**B**) The protein-protein docking was performed using the ClusPro 2.0 web server. Model exhibiting the probable interaction of nsP2 (red surface, PDB ID: 3TRK) with Catenin-α-1 (green surface, PDB ID: 4IGG). (**C**)The figure highlights polar interactions (yellow bridge) between residues of nsP2 (red) and host Catenin-α-1 (green).

**Fig 2:**
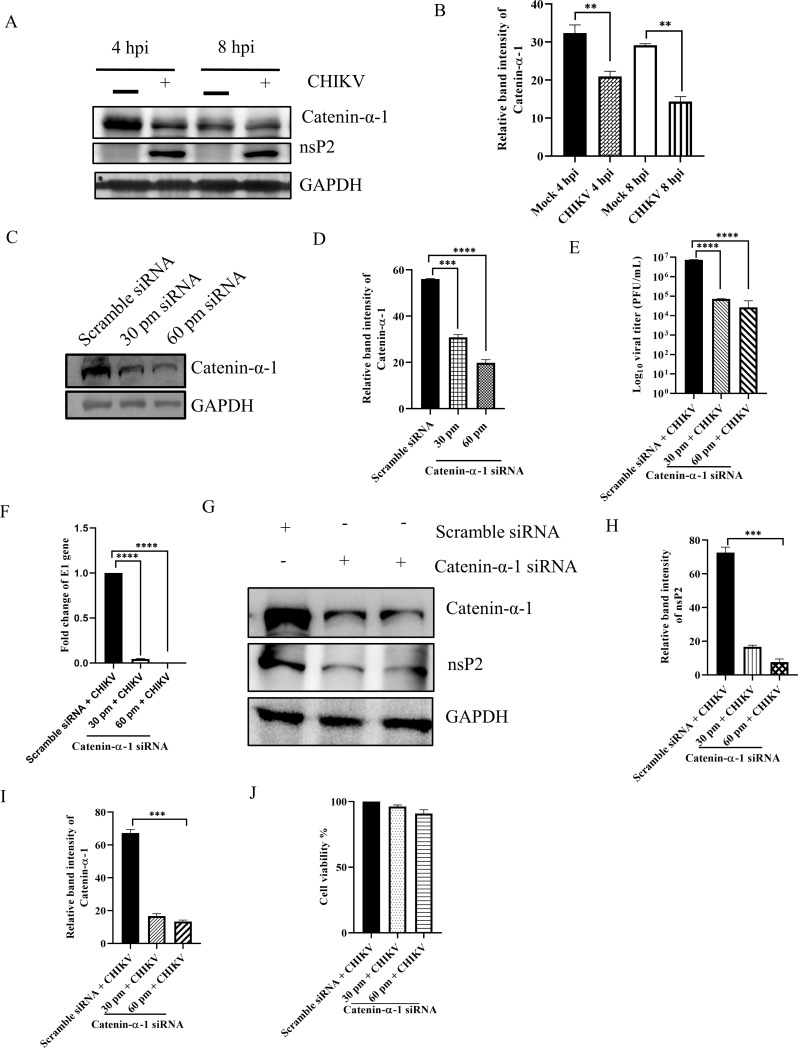
The Catenin-α-1 protein is crucial for CHIKV infection. Vero cells were mock-infected or infected with CHIKV and harvested at various time points. (**A**) Western blot was performed using nsP2, Catenin-α-1, and GAPDH antibodies. (**B**) Bar diagram showing relative band intensity of Catenin-α-1 in Vero cells at different times post infection. (**C**) The HEK293T cells were transfected with scramble siRNA or 30 and 60 pm of Catenin-α-1 siRNA. Catenin-α-1 level was estimated by Western blot, and GAPDH was used as a loading control. (**D**) Bar diagram showing relative band intensity of the Catenin-α-1 protein following Catenin-α-1 siRNA transfection in HEK293T cells. (**E**) After 24 hours post-transfection (hpt), cells were infected with CHIKV (MOI = 0.1) and harvested at 15 hpi for further downstream experiments. Bar diagram representing the Log_10_ of viral titer in the cell supernatant of scramble + CHIKV and 30 and 60 pm Catenin-α-1 siRNA + CHIKV samples in HEK293T cells. (**F**) Total RNA was isolated from the scramble + CHIKV and 30 and 60 pm Catenin-α-1 siRNA + CHIKV samples, and the CHIKV-E1 gene was amplified by RT-qPCR. Bar diagram displaying the fold changes of viral RNA in HEK293T cells. (**G**) Western blot exhibiting the nsP2 and Catenin-α-1 protein levels after transfection and infection with CHIKV in HEK293T cells. (**H**) and (**I**) Bar diagrams depicting relative band intensities of the nsP2 and Catenin-α-1 proteins. Data from three independent experiments are shown as mean ± SD, and the One-way ANOVA test was used for all the comparisons. **P ≤ 0.01; ***P ≤ 0.001; and ****P ≤ 0.0001 were considered statistically significant.

A wide array of host factors, in addition to the non-structural proteins, leads to effective CHIKV infection (1, 2). However, there is still much to explore on how cellular proteins contribute to effective CHIKV infection. At present, neither treatments nor vaccines are available for CHIKV infection, making it a public health threat. The lack of effective remedies against this virus is partly a result of information gaps about the basic mechanisms of viral infection and interactions between CHIKV and host proteins. Mass spectrometry data from our lab revealed that Catenin-α-1 was interacting with CHIKV-nsP2 (Table S1). Hence, it was crucial to ascertain the identification of these cellular proteins like Catenin-α-1 and comprehend their specific contribution to CHIKV infection. In order to validate the interaction between Catenin-α-1 and CHIKV-nsP2, Vero cells were infected with CHIKV and harvested at 6 hours post-infection (hpi) and subsequently subjected to co-immunoprecipitation and Western blot analysis (1, 3). The results demonstrated that Catenin-α-1 effectively immunoprecipitated the CHIKV-nsP2 protein, while IgG was used as a negative control. Further experiments unveiled that CHIKV-nsP3 does not interact with Catenin-α-1, demonstrating the specificity of the interaction between Catenin-α-1 and CHIKV-nsP2 (Fig. 1A). To unravel the potential amino acid residues responsible for this interaction, protein-protein docking was carried out. Close interaction was predicted between Catenin-α-1 (PDB ID: 4IGG) and nsP2 (PDB ID: 3TRK) with multiple polar interactions as shown in Fig. 1B and C. Together, the data indicate that Catenin-α1 interacts with CHIKV-nsP2 during CHIKV infection. To assess the level of Catenin-α-1 during CHIKV infection, Vero cells were infected with CHIKV at an MOI of 2, and cells were harvested at different time points followed by Western blot. The outcome showed that Catenin-α-1 was downregulated in CHIKV-infected cells compared to the mock cells (Fig. 2A and B). Next, in order to understand the role of Catenin-α-1 in CHIKV infection, the gene was silenced using 30 and 60 pm concentrations of Catenin-α1 siRNA in the HEK293T cells.

After 24 hpt, cells were harvested and subjected to Western blot analysis. The results showed a decrease of 45% and 64.6% in the levels of Catenin-α-1 using 30 and 60 pm concentrations of siRNA, respectively, compared to the scramble siRNA control (Fig. 2C and D). Subsequently, the siRNA-transfected cells (30 and 60 pm siRNA) were infected with CHIKV, and the supernatant and cells were processed for plaque assay, total RNA isolation, and Western blot analysis. It was found to have more than 90% reduction (95% and 99%) in the viral particle formation and in the level of E1 gene after 30 and 60 pm of siRNA transfection, compared to the scramble control (Fig. 2E and F). Furthermore, Western blot analysis demonstrated a substantial and dose-dependent reduction (77.1% and 89.5%) in the nsP2 protein level following siRNA transfection (Fig. 2G and H). Catenin-α-1 level was also reduced significantly (Fig. 2G and I). Additionally, the cell viability under the knock-down conditions revealed that 90% of the cells were viable under infected knock-down conditions (Fig. 2J). Collectively, these results indicate the vital role of Catenin-α-1 for effective CHIKV infection.

A previous report suggested that mutation in Catenin-α-1 leads to familial exudative vitreoretinopathy, a severe retinal vascular disease (4). Another report indicated that the loss of functional α- and β-catenin contributes to the progression and invasion of lobular carcinoma to lobular invasive carcinoma *in situ* (5). However, this protein has never been explored in the case of any virus, making the current study novel by demonstrating the crucial role of Catenin-α-1 in the case of CHIKV.

Cell adhesion and the reorganization of the actin cytoskeleton were mediated by Catenin-α-1 in the case of cancer (6), and CHIKV is also known to promote actin polymerization (7). Hence, CHIKV might modulate Catenin-α-1 (which is a part of the membrane adhesion complex) for efficient infection. Nevertheless, the precise mechanism by which Catenin-α-1 is regulated during viral infection remains unexplored and requires further investigation. In conclusion, the current investigation identified Catenin-α-1 as an essential host factor in CHIKV infection, which could aid in designing effective therapeutics to control this infection in the future.

## Data Availability

The data that support the findings of this study are available from the corresponding author upon reasonable request. Some data may not be made available because of privacy or ethical restrictions.

## SUPPLEMENTAL MATERIAL

Table S1 spectrum.02485-23-s0001.docx Interacting partners of CHIKV-nsP2 using mass spectrometry.

ASM does not own the copyrights to Supplemental Material that may be linked to, or accessed through, an article. The authors have granted ASM a non-exclusive, world-wide license to publish the Supplemental Material files. Please contact the corresponding author directly for reuse.
