# Supplementary material for: A hidden gem Catenin-α-1 is essential for Chikungunya virus infection
Source: Microbiol Spectr. 2023 Nov 14;11(6):e02485-23. doi: 10.1128/spectrum.02485-23 (PMC10715081; doi:10.1128/spectrum.02485-23)
Supplement: Table S1 — Interacting partners of CHIKV-nsP2 using mass spectrometry. [file spectrum.02485-23-s0001.docx]

Supplementary Material

**A hidden gem Catenin-α-1 is essential for Chikungunya virus infection**

**Sanchari Chatterjee^1,2^, Bharat Bhusan Subudhi^3^, Soma Chattopadhyay^1*^**

*** Correspondence: Dr. Soma Chattopadhyay**

# Supplementary Table

Table S1. Table depicting interacting partners of CHIKV-nsP2 using Mass spectrometry

| Sl No. | Protein name | Species Name | Mol.Wt (kDa) |
| --- | --- | --- | --- |
| 1 | KIAA1631 protein | [Homo sapiens] | 110 |
| 2 | Catenin-α-1 | [Mus musculus] | 100 |
| 3 | 55.11 protein homolog - human (fragment) | [Homo sapiens] | 100 |
| 4 | Elongation factor 2 | [Homo sapiens] | 100 |
| 5 | Importin subunit beta-1 | [Mus musculus] | 100 |
| 6 | Heat shock protein 90 | [Homo sapiens] | 85 |
| 7 | Glycyl-tRNA synthetase | [Homo sapiens] | 85 |
| 8 | Putative pre-mRNA-splicing factor ATP-dependent RNA helicase DHX15 isoform 2 | [Mus musculus] | 85 |
| 9 | Stress-70 protein, mitochondrial | [Mus musculus] | 80 |
| 10 | Nucleolin | [Homo sapiens] | 80 |
| 11 | Heterogeneous nuclear ribonucleoprotein M isoform a | [Mus musculus] | 80 |
| 12 | BBC1 | [Homo sapiens] | 20 |
